# Supplementary material for: Mice Carrying ALS Mutant TDP-43, but Not Mutant FUS, Display In Vivo Defects in Axonal Transport of Signaling Endosomes
Source: Cell Rep. 2020 Mar 17;30(11):3655–3662.e2. doi: 10.1016/j.celrep.2020.02.078 (PMC7090381; doi:10.1016/j.celrep.2020.02.078)
Supplement: Document S1. Figures S1–S4 and Table S1 [file mmc1.pdf]

Cell Reports, Volume *30*

Supplemental Information

Mice Carrying ALS Mutant TDP-43, but Not Mutant FUS, Display *In Vivo* Defects in Axonal Transport of Signaling Endosomes

James N. Sleigh, Andrew P. Tosolini, David Gordon, Anny Devoy, Pietro Fratta, Elizabeth M.C. Fisher, Kevin Talbot, and Giampietro Schiavo

Supplemental Figures

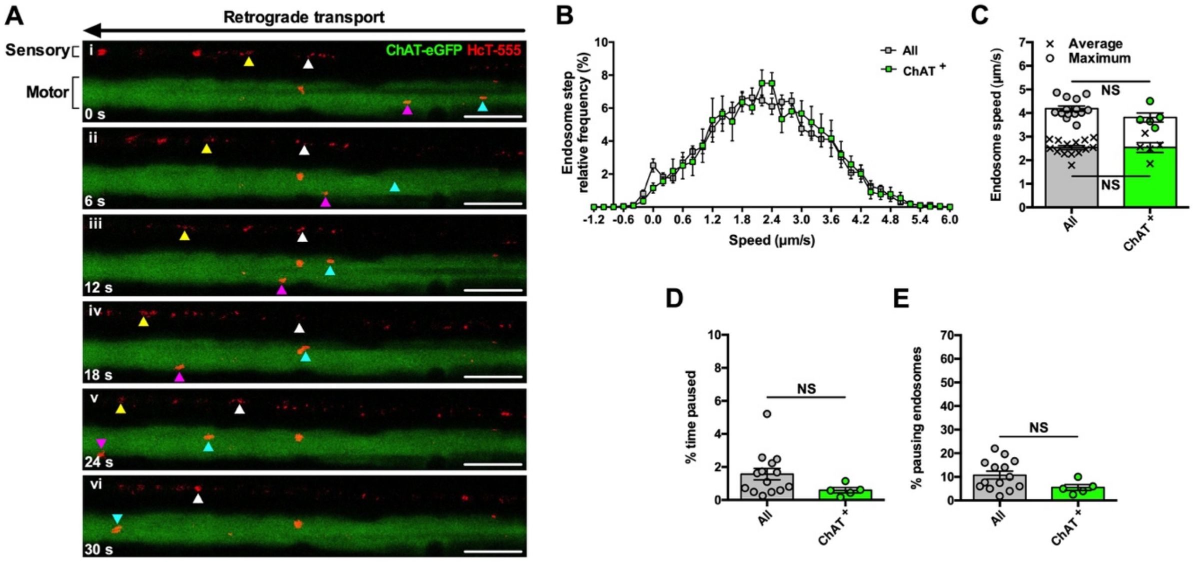

Figure S1. Signalling endosome dynamics are similar between ChAT⁺ axons and the neurons assessed in non-fluorescent animals. Related to Figure 1. (**A**) Individual signalling endosomes loaded with fluorescently labelled HcT (HcT-555, red) can be imaged in live, intact sciatic nerve axons using time-lapse confocal microscopy and their dynamics subsequently assessed. This series of images (i-vi) depicts retrograde (right to left) axonal transport of distinct endosomes identified by coloured arrowheads. These images were taken from a ChAT-eGFP mouse, which allows the differentiation between motor (ChAT-eGFP$^+$, green) and sensory (ChAT-eGFP$^-$) axons. Note how much thinner the sensory axons are than the motor axons, causing endosome transport in sensory nerves to occur in a narrow, straight line. Scale bars = 10 µm. (**B**) The speed distribution curve of signalling endosome frame-to-frame movements in ChAT$^+$ motor neurons (green) is similar to that generated from all axons of 3 month old wild-type, non-fluorescent mice (All, grey). This suggests that motor neurons are predominantly assessed throughout this study. (**C-E**) There is no difference between ChAT$^+$ motor neurons and axons from 3 month old, non-fluorescent wild-type mice in mean endosome speeds (crosses, C), maximum endosome speeds (circles, C), percentage time paused (D), or the percentage of endosomes that paused (E). The ChAT$^+$ data presented here are the same as that in **Figure 1**. NS, not significant, unpaired *t*-test. *n* = 5-14. Means ± SEM are plotted for all graphs.

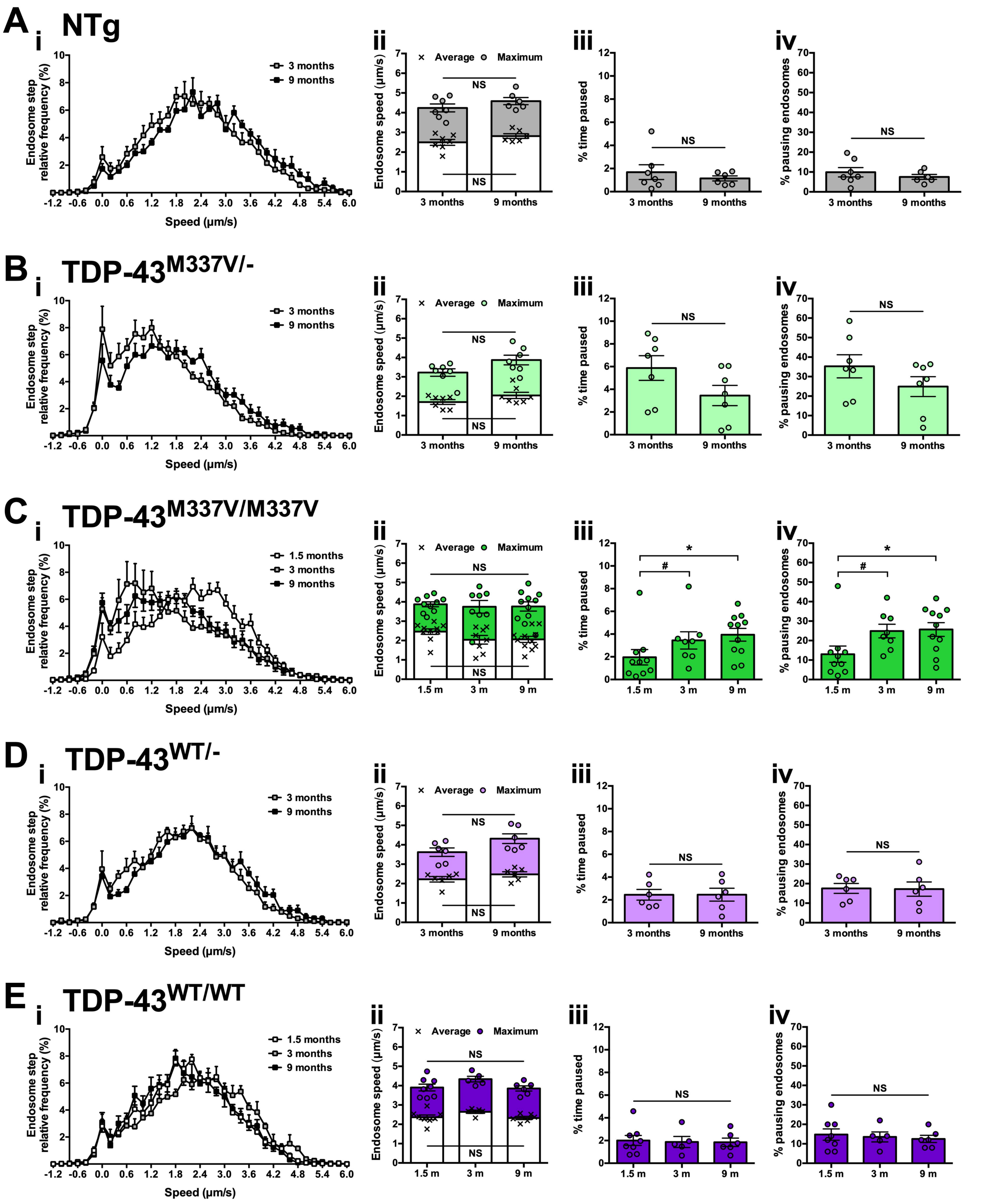

Figure S2. The TDP-43^{M337V} transport defect presents between 1.5 and 3 months and persists thereafter. Related to Figures 2 and 3. (**A-E**) Axonal signalling endosome kinetics do not change significantly from 3 to 9 months in NTg (A), TDP-43$^{M337V/-}$ (B), and TDP-43$^{WT/-}$ (D) mice or from 1.5 to 9 months in TDP-43$^{WT/WT}$ animals (E); however, signalling endosome transport becomes impaired in TDP-43$^{M337V/M337V}$ mice between 1.5 and 3 months (C, ii $P = 0.143$; iii $P = 0.0273$; iv $P = 0.0209$, Kruskal-Wallis test). The data presented in this figure are also found in **Figures 2** and **3**. $^{\#}$ $P < 0.05$; Mann-Whitney U test; * $P < 0.05$; Dunn's multiple comparisons test. NS, not significant, unpaired t-test/Mann Whitney U test/one-way ANOVA. n = 5-11. Means ± SEM are plotted for all graphs.

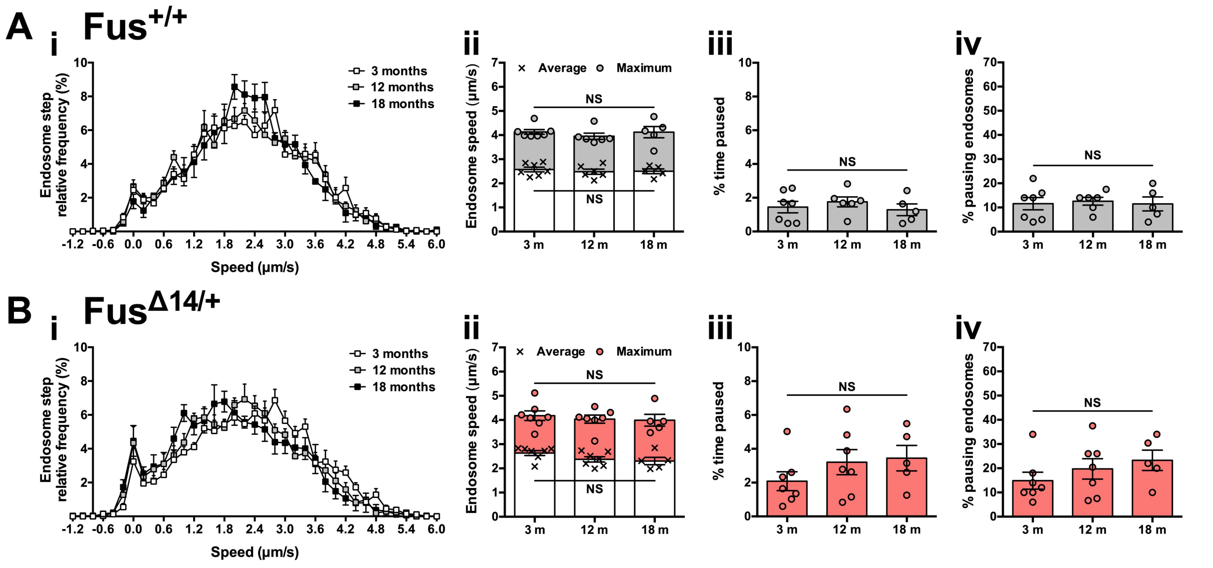

Figure S3. Fus$^{\Delta 14/+}$ axonal transport remains largely unchanged between 3 and 18 months. Related to Figure 4. (**A** and **B**) Endosome axonal dynamics do not significantly change from 3 to 18 months in Fus$^{+/+}$ (A) and Fus$^{\Delta 14/+}$ (B) mice. However, there is a minor, progressive decline in endosomal dynamics in mutant Fus mice. The data presented in this figure are also found in **Figure 4**. NS, not significant, one-way ANOVA. *n* = 5-7. Means ± SEM are plotted for all graphs.

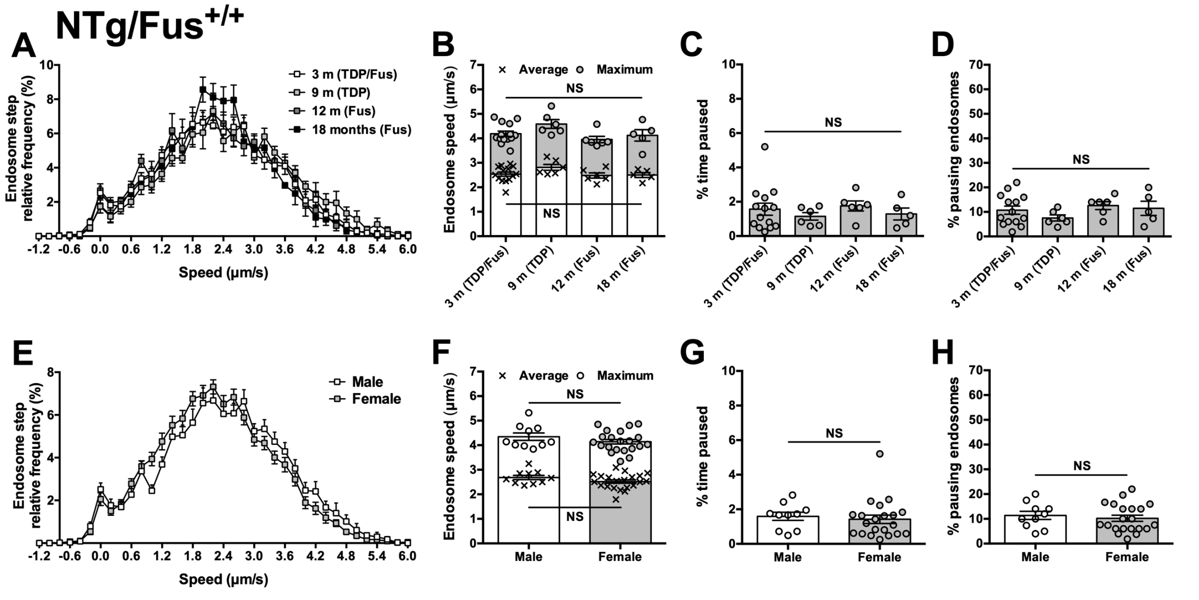

Figure S4. Axonal endosome kinetics do not change from 3 to 18 months or differ between sexes in wild-type mice. Related to Figures 2-4 (**A-D**) There is no difference in signalling endosome dynamics between wild-type mice aged 3, 9, 12, and 18 months, indicating that aging of mice to 18 months does not affect axonal transport of signalling endosomes. Data generated from 3 month old wild-type mice, used as controls for TDP-43^{M337V} and Fus$^{\Delta 14/+}$, were combined as they showed no significant difference (not shown). (**E-H**) There is no difference in axonal kinetics of signalling endosomes between male and female mice. Since there was no difference between timepoints (A-D), combined data from all ages are presented. The data presented in this figure are also found in **Figures 2-4**. NS, not significant, one-way ANOVA. *n* = 5-14 (A-D) and 10-21 (E-H). Means ± SEM are plotted for all graphs.

Supplemental Tables

ALS mouse model	*In vivo* axonal transport	Lumbar motor neuron loss
$SOD1^{G93A}$ [(1)]	Motor neuron-specific, pre-symptomatic (P36 onwards, and worst at P73) reduction in retrograde transport speed of signalling endosomes in sciatic nerve axons (only females tested) [(2-4)]<br><br>Pre-symptomatic (P36) increase in anterograde and retrograde mitochondrial pausing resulting in reduced speeds in sciatic nerve axons (only females tested) [(2)]<br><br>Reduced retrograde mitochondrial movement in sciatic nerve axons at P45, but not P15, progressively worsening and also affecting anterograde transport by P90 (males and females) [(5)]	0% at P36;<br>23% at P73;<br>39% at P94;<br>56% at P113 [(2)]
Prp-$TDP\text{-}43^{A315T}$ [(6)]	Reduced anterograde and retrograde mitochondrial movement in sciatic nerve axons at P90, but not P45 (males and females) [(5)]	20% at end stage [(6)]
$TDP\text{-}43^{M337V}$ [(7)]	Pre-symptomatic (3 and 9 months, but not 1.5 months) reduction in retrograde transport speed of signalling endosomes in sciatic nerve axons, at least in part, through increased pausing (males and females) [(8)]	0% at 12 months [(7)]
$Fus^{\Delta 14/+}$ [(9)]	Retrograde transport of signalling endosomes in sciatic nerve axons unaffected at 3 and 12 months, with only a minor increase in pausing at 18 months, which does not affect transport speed (males and females) [(8)]	0% at 3 months;<br>14% at 12 months;<br>20% at 18 months [(9)]

Table S1. *In vivo* axonal transport phenotypes and motor neuron loss in mouse models of ALS. Related to Figures 2-4. *In vivo* axonal transport defects, as assessed by individual cargo tracking, have now been reported in a number of mouse models of ALS. For comparison purposes, this table provides details on the axonal trafficking disruption and lumbar spinal cord motor neuron loss. *N.B.*, *in vivo* studies in which transport has been assessed *en masse* are not included. [(1)] Gurney et al., 1994; [(2)] Bilsland et al., 2010; [(3)] Gibbs et al., 2018; [(4)] Fellows et al., 2020; [(5)] Magrané et al., 2014; [(6)] Wegorzewska et al., 2009; [(7)] Gordon et al., 2019; [(8)] this study; [(9)] Devoy et al., 2017.
