# Supplementary material for: Mice Carrying ALS Mutant TDP-43, but Not Mutant FUS, Display In Vivo Defects in Axonal Transport of Signaling Endosomes
Source: Cell Rep. 2020 Mar 17;30(11):3655–3662.e2. doi: 10.1016/j.celrep.2020.02.078 (PMC7090381; doi:10.1016/j.celrep.2020.02.078)
Supplement: Document S2. Article plus Supplemental Information [file mmc2.pdf]

Cell Reports

Report

Mice Carrying ALS Mutant TDP-43, but Not Mutant FUS, Display *In Vivo* Defects in Axonal Transport of Signaling Endosomes

Graphical Abstract

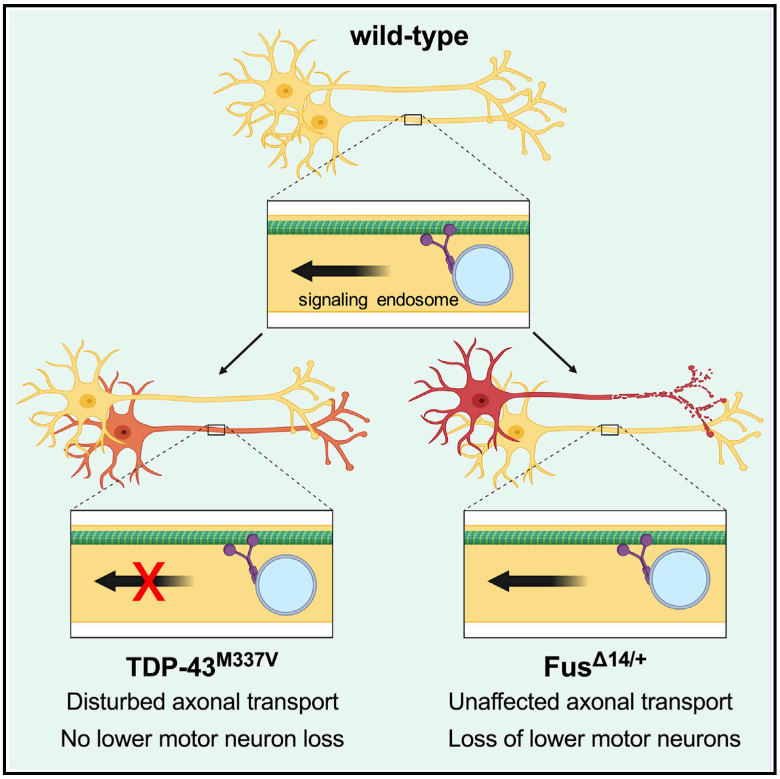

Highlights

- Mutant TDP-43 mice display *in vivo* defects in signaling endosome axonal transport
- Despite motor neuron loss, mutant FUS mice display no such deficiency
- Axonal transport disruption is thus not common to all ALS mouse models

Authors

James N. Sleigh, Andrew P. Tosolini, David Gordon, ..., Elizabeth M.C. Fisher, Kevin Talbot, Giampietro Schiavo

Correspondence

j.sleigh@ucl.ac.uk (J.N.S.),
giampietro.schiavo@ucl.ac.uk (G.S.)

In Brief

Sleigh et al. address the importance of disturbances in axonal transport in two mouse models for ALS. They find that deficiencies in the *in vivo* axonal delivery of target tissue-derived survival factors are not a common feature of all ALS mouse models, suggesting mechanistic distinctions in different ALS-linked genes.

Sleigh et al., 2020, Cell Reports *30*, 3655–3662
March 17, 2020 © 2020 The Author(s).
https://doi.org/10.1016/j.celrep.2020.02.078

CellPress

Cell Reports
Report

OPEN ACCESS
CellPress

Mice Carrying ALS Mutant TDP-43, but Not Mutant FUS, Display *In Vivo* Defects in Axonal Transport of Signaling Endosomes

James N. Sleigh,[1,2,*] Andrew P. Tosolini,[1] David Gordon,[3] Anny Devoy,[1,5] Pietro Fratta,[1] Elizabeth M.C. Fisher,[1] Kevin Talbot,[3] and Giampietro Schiavo[1,2,4,6,*]
[1]Department of Neuromuscular Diseases, UCL Queen Square Institute of Neurology, University College London, London WC1N 3BG, UK
[2]UK Dementia Research Institute, University College London, London WC1E 6BT, UK
[3]Nuffield Department of Clinical Neurosciences, University of Oxford, John Radcliffe Hospital, Oxford OX3 9DU, UK
[4]Discoveries Centre for Regenerative and Precision Medicine, University College London Campus, London WC1N 3BG, UK
[5]Present address: UK Dementia Research Institute, Maurice Wohl Clinical Neuroscience Institute, Kings College London, London SE5 9RX, UK
[6]Lead Contact
*Correspondence: j.sleigh@ucl.ac.uk (J.N.S.), giampietro.schiavo@ucl.ac.uk (G.S.)
https://doi.org/10.1016/j.celrep.2020.02.078

SUMMARY

Amyotrophic lateral sclerosis (ALS) is a fatal, progressive neurodegenerative disease resulting from a complex interplay between genetics and environment. Impairments in axonal transport have been identified in several ALS models, but *in vivo* evidence remains limited, thus their pathogenetic importance remains to be fully resolved. We therefore analyzed the *in vivo* dynamics of retrogradely transported, neurotrophin-containing signaling endosomes in nerve axons of two ALS mouse models with mutations in the RNA processing genes *TARDBP* and *FUS*. TDP-43^{M337V} mice, which show neuromuscular pathology without motor neuron loss, display axonal transport perturbations manifesting between 1.5 and 3 months and preceding symptom onset. Contrastingly, despite 20% motor neuron loss, transport remained largely unaffected in Fus$^{\Delta14/+}$ mice. Deficiencies in retrograde axonal transport of signaling endosomes are therefore not shared by all ALS-linked genes, indicating that there are mechanistic distinctions in the pathogenesis of ALS caused by mutations in different RNA processing genes.

INTRODUCTION

Amyotrophic lateral sclerosis (ALS) is a rapidly progressive neurodegenerative disorder that results from upper and lower motor neuron loss, leading to muscle wasting, atrophy, and, ultimately, death most often due to respiratory failure (Brown and Al-Chalabi, 2017). Treatment options for ALS patients are severely limited, but gene therapy approaches hold great promise (Tosolini and Sleigh, 2017). ALS is thought to manifest through a multi-step process encompassing additive effects from genetic predispositions and environmental insults (Al-Chalabi and Hardiman, 2013); however, ∼10% of cases show clear monogenic heritability (familial ALS [fALS]), while known causative genetic mutations underlie ∼68% of fALS and ∼11% of the remaining sporadic cases of ALS (Renton et al., 2014). Mutations in numerous genes are linked to the disease, the four most common of which, in ascending order, are dominant mutations in fused in sarcoma (*FUS*), transactive-region DNA-binding protein (*TARDBP* encoding TDP-43), superoxide dismutase 1 (*SOD1*), and large, intronic hexanucleotide repeat expansions in chromosome 9 open reading frame 72 (*C9orf72*) (Brown and Al-Chalabi, 2017).

Many genes associated with ALS encode proteins important in all cells, and as such, it remains unknown why motor neurons and certain brain regions, such as the frontotemporal cortex, are selectively affected. Nonetheless, impairments in cytoskeletal dynamics and axonal transport are emerging as a central theme based on ALS-linked gene function (Clark et al., 2016; De Vos and Hafezparast, 2017; Sleigh et al., 2019). Axonal transport is the fundamental, bi-directional process whereby cargoes (e.g., organelles and proteins) are actively transported from one end of an axon to the other, along polarized microtubules (Maday et al., 2014). Anterograde transport, which is from the cell body to axon terminal, is dependent on the kinesin family of molecular motors, while the cytoplasmic dynein complex is responsible for retrograde axonal transport in the opposite direction. Patient post-mortem studies provided the first evidence for involvement of impaired transport in ALS, which has since been consolidated by results from a plethora of disease models implicating various cargoes (De Vos and Hafezparast, 2017). Transport deficits have been linked to all four major ALS genes through *in vitro*, *ex vivo*, and *Drosophila melanogaster* experiments; however, these experimental models do not necessarily replicate the complex environment found in mammals, which is required for efficient, rapid axonal transport (Sleigh et al., 2017). *In vivo* results from mammals in which individual cargoes are tracked in real time, rather than en masse, have been generated in SOD1^{G93A} and TDP-43^{A315T} ALS mice (Table S1; Bilsland et al., 2010; Fellows et al., 2020; Gibbs et al., 2018; Magrané et al., 2014). Axonal transport is disrupted in both models at early disease stages,

This is an open access article under the CC BY license (http://creativecommons.org/licenses/by/4.0/).

OPEN
ACCESS
CellPress

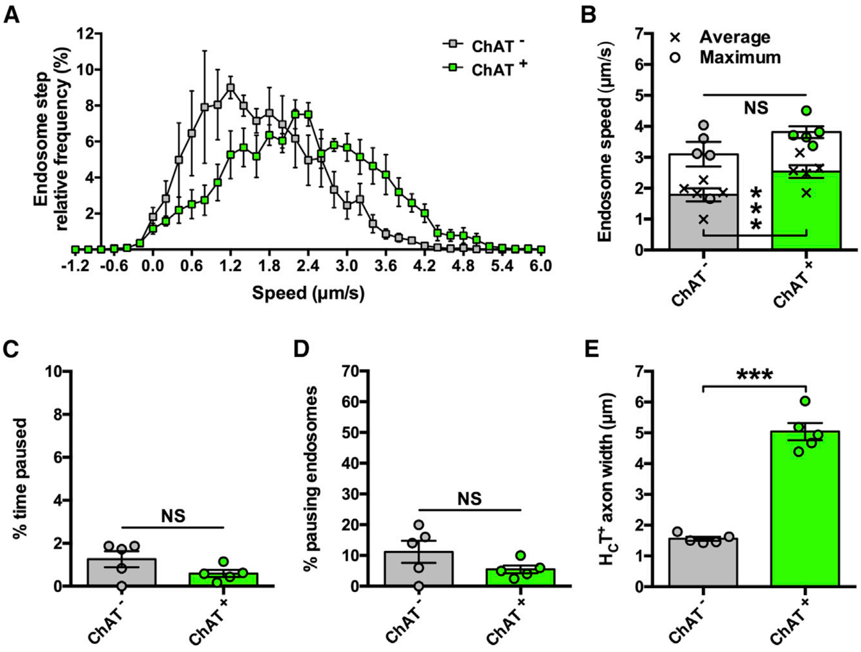

Figure 1. Retrograde Axonal Transport of Signaling Endosomes Is Faster in Motor Neurons Than Sensory Neurons

(A) Speed distribution curves of signaling endosome frame-to-frame movements in motor ($ChAT^+$, green) and sensory axons ($ChAT^-$, gray) indicate that axonal transport is faster in motor neurons.

(B) Mean (crosses), but not maximum (circles), endosome transport is faster in motor neurons when calculated per animal.

(C and D) There is no difference between motor and sensory nerves in the percentage of time endosomes paused for (C) or the percentage of endosomes that paused (D).

(E) H_CT-containing axons that are $ChAT^+$ have a larger caliber than $ChAT^-$ axons.

***$p < 0.001$ and NS, not significant, paired t test. n = 5. Means ± standard error of the mean (SEM) plotted for all graphs.

See also Figure S1.

consistent with a potential causative role in neuromuscular dysfunction and motor neuron degeneration; nonetheless, it is unclear whether these ALS mice, which express disease-causing mutant proteins at supra-physiological levels, are reflective of the full disease spectrum.

We have thus performed pseudolongitudinal assessments of *in vivo* axonal transport in two recently engineered mouse models of ALS with mutations in genes encoding DNA/RNA-binding proteins instrumental to RNA processing, TDP-43 (Gordon et al., 2019) and Fus (Devoy et al., 2017). Transgenic $TDP\text{-}43^{M337V}$ and humanized, knockin $Fus^{\Delta 14/+}$ mice, which both express mutant protein at physiologically relevant levels, have been used to address the importance of altered axonal transport to ALS neuropathology. Although mitochondria are the most frequently analyzed axonal cargo, in this study we opted to assess the trafficking of signaling endosomes, which are essential to the long-range delivery of signals critical to neuronal survival.

RESULTS

Imaging *In Vivo* Axonal Transport in Motor Neurons

To assess *in vivo* dynamics of axonal transport, we used a fluorescently labeled, binding fragment of tetanus neurotoxin (H_CT), which is retrogradely transported along axons within neurotrophin-containing signaling endosomes toward neuronal cell bodies (Surana et al., 2018; Villarroel-Campos et al., 2018). Impairments in long-range neurotrophic signaling have been implicated in several neurodegenerative conditions, including ALS (Bronfman et al., 2007; Sleigh et al., 2019). By injecting H_CT into the gastrocnemius and tibialis anterior muscles of the lower leg, and exposing the sciatic nerve at mid-thigh level 4–8 h post-injection, individual, fluorescently labeled endosomes being retrogradely transported can be imaged and tracked in the peripheral nerve axons of live, anesthetized mice (Figure S1A; Gibbs et al., 2016; J.N.S., A.P.T., and G.S., unpublished data).

Post-intramuscular injection, about 80% of H_CT-positive (H_CT^+) axons stain for choline acetyltransferase (ChAT) (Bilsland et al., 2010), suggesting that the probe is preferentially transported in motor neurons. Nevertheless, assessing transport in a mixed motor and sensory population may weaken the ability to identify motor-specific trafficking perturbations. Therefore, before analyzing transport in ALS mice, we compared endosome dynamics in motor versus sensory neurons using ChAT-eGFP mice, which permit visual differentiation of peripheral nerve types because motor axons are specifically labeled with eGFP (Figure S1A). Mean endosome transport speeds were greater in $ChAT^+$ motor neurons compared with $ChAT^-$ sensory neurons (Figures 1A and 1B), and this was not due to major pausing differences (Figures 1C and 1D). Interestingly, motor axons had clearly larger calibers than sensory axons (Figure 1E), suggesting that by imaging thicker axons, H_CT transport can be measured in motor neurons with greater certainty than if randomly selecting an axon (i.e., >80%; Bilsland et al., 2010). To confirm this, transport dynamics were compared between $ChAT^+$ axons and thicker axons from non-fluorescent control mice, and no differences were observed (Figure S1). The bell-shaped, rather than bi-modal, speed frequency distribution generated from non-fluorescent mice (Figure S1B) indicates that *in vivo* axonal transport of endosomes can be assessed predominantly in motor neurons by selecting large-caliber axons. This approach was thus used to analyze axonal transport in ALS mice.

In Vivo Axonal Transport Is Pre-symptomatically Impaired in Mutant TDP-43 Mice

Recently reported transgenic $TDP\text{-}43^{M337V}$ mice display impairments in motor function and neuromuscular junction abnormalities beginning at 9 months in homozygous mutants without

OPEN
ACCESS
CellPress

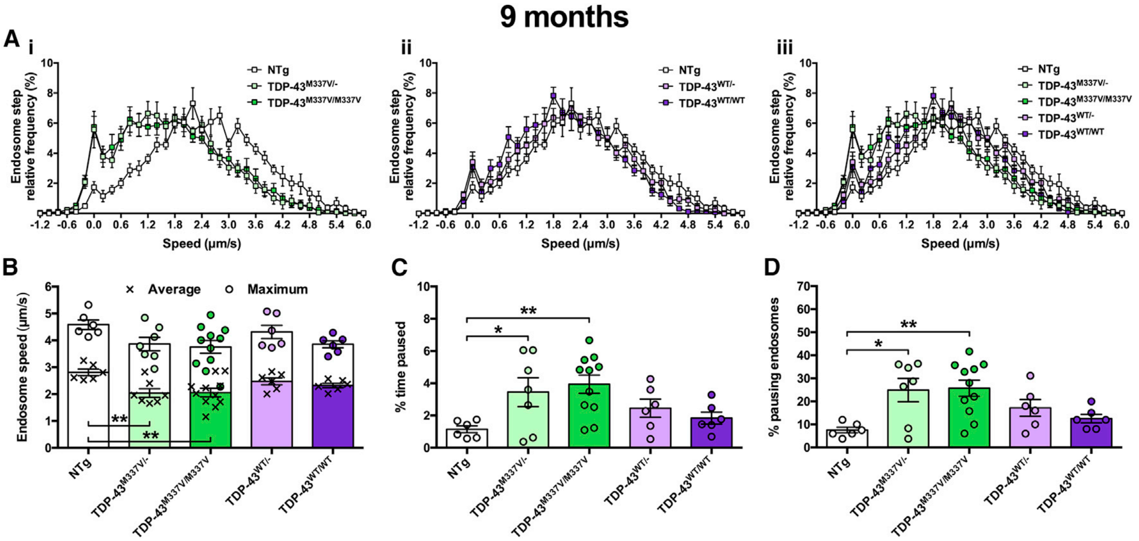

Figure 2. Mutant TDP Mice Display Perturbed Axonal Transport of Endosomes at 9 Months

(A) Endosome frame-to-frame speed distribution curves indicate that hemizygous and homozygous TDP-43^{M337V} mice (i, iii, green) transport endosomes more slowly than control, non-transgenic (NTg) mice (white), whereas transport is unaffected in TDP-43^{WT} controls (ii, iii, purple).

(B) Mean endosomal speeds (crosses) are significantly reduced in TDP-43$^{M337V/-}$ and TDP-43$^{M337V/M337V}$ mice, but not TDP-43^{WT} controls ($p = 0.006$, one-way ANOVA), while maximum speeds (circles) remained unchanged ($p = 0.0891$, one-way ANOVA).

(C) Endosomes in mutant TDP-43^{M337V} hemizygous and homozygous mice paused for longer periods of time compared with NTg mice ($p = 0.0139$, one-way ANOVA).

(D) TDP-43^{M337V} mice had a greater percentage of endosomes that paused ($p = 0.0046$, one-way ANOVA).

*$p < 0.05$ and **$p < 0.01$, Dunnett's multiple-comparisons test. n = 6–11. Means ± SEM are plotted for all graphs.

See also Figures S2 and S4.

motor neuron loss up to 12 months (Gordon et al., 2019; Williamson et al., 2019). We therefore first assessed retrograde transport of signaling endosomes at 9 months of age in hemizygous and homozygous TDP-43^{M337V} and TDP-43^{WT} mice and non-transgenic (NTg) controls (Figure 2). The frequency histograms of frame-to-frame endosome speeds of both TDP-43$^{M337V/-}$ and TDP-43$^{M337V/M337V}$ animals are shifted to the left compared with NTg mice, indicative of slower transport, whereas TDP-43^{WT} transport was unaffected because it overlaps with the curve obtained using NTg controls (Figure 2A). When compared, both mutants showed a statistically significant reduction in mean endosome speed (Figure 2B), which was at least partially due to increased pausing (Figures 2C and 2D). Mutant TDP-43 mice do not show clear behavioral phenotypes at 3 months (Gordon et al., 2019; Williamson et al., 2019); we therefore assessed transport at this early time point to see whether axonal transport defects precede symptom onset and thus may contribute to motor neuron pathology. Indeed, a similar deficiency in mutant TDP-43 transport was observed at 3 months, while TDP-43^{WT} transport remained unperturbed (Figures 3A–3D). Finally, to determine at what stage transport becomes affected, we assessed endosomal trafficking at 1.5 months in TDP-43$^{M337V/M337V}$ and TDP-43$^{WT/WT}$ mice. We found no difference between genotypes (Figures 3E–3H) or from NTg control mice (not shown). These data indicate that TDP-43^{M337V}, but not TDP-43^{WT}, mice display a pre-symptomatic, non-developmental *in vivo* impairment in axonal transport of signaling endosomes that manifests between 1.5 and 3 months of age (Figure S2).

Axonal Transport Remains Largely Unaffected in Mutant Fus Mice Even at Late Stages

Deficient *in vivo* trafficking of signaling endosomes has now been observed in SOD1^{G93A} mice (Bilsland et al., 2010; Gibbs et al., 2018) and the TDP-43^{M337V} model reported here. To assess whether this phenotype is common to mouse models of ALS, we assessed *in vivo* transport in knockin mutant Fus$^{\Delta14/+}$ mice. This model displays loss of neuromuscular integrity and progressive degeneration of lumbar spinal motor neurons; at 3 months, mutant Fus mice show no motor neuron loss, which becomes overt by 12 (14% reduction) and 18 (20% reduction) months of age (Devoy et al., 2017). We therefore assessed endosome transport at 3, 12, and 18 months in this ALS model (Figure 4). At 3 and 12 months, there was no significant difference in axonal kinetics of these organelles (Figures 4A–4H), and, despite an increase in pausing (Figures 4K and 4L), there was no significant change in signaling endosome mean or maximum speeds at the late disease stage of 18 months (Figures 4I and 4J). Consistent with this, no significant changes in transport were observed across time points for Fus$^{+/+}$ or Fus$^{\Delta14/+}$ mice, although Fus mutants show a subtle, progressive, yet non-significant, decline (Figure S3).

We have previously shown that endosome transport remains stable in wild-type mice from 1 to 13–14 months of age (Sleigh and Schiavo, 2016), suggesting that a natural, aging-related decline in transport does not compound the mutant TDP-43 transport defect. To ensure that this remains true up to 18 months, we compared axonal transport in all control mice

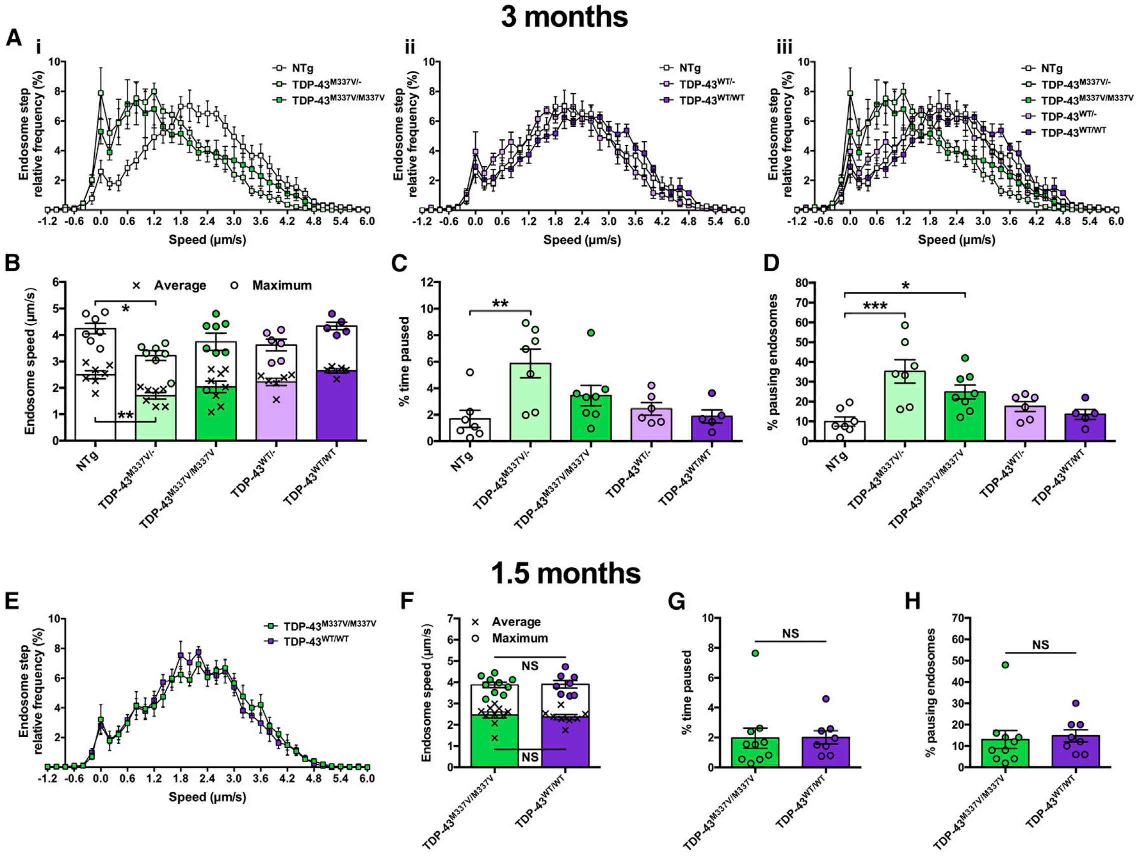

Figure 3. TDP-43^{M337V} Transport Disruption Occurs between 1.5 and 3 Months

(A) Endosome frame-to-frame speed distribution curves show that at 3 months TDP-43^{M337V} (i, iii, green), but not TDP-43^{WT} (ii, iii, purple), mice transport endosomes more slowly than control, non-transgenic (NTg) mice (white).

(B) Mean (crosses, $p = 0.004$, one-way ANOVA) and maximum (circles, $p = 0.0222$, one-way ANOVA) endosomal speeds are significantly reduced in TDP-43$^{M337V/-}$ mice.

(C and D) TDP-43^{M337V} hemizygotes and homozygotes show increased endosome pausing as assessed by calculating the percentage of time paused (C) ($p = 0.0171$, Kruskal-Wallis test) and the percentage of pausing endosomes (D) ($p < 0.001$, one-way ANOVA).

(E–H) At 1.5 months, there is no difference in endosome frame-to-frame speed distribution curves (E), average or maximum endosome transport speeds (F), the percentage of time that endosomes were paused (G), or the percentage of pausing endosomes (H) between TDP-43$^{M337V/M337V}$ (green) and TDP-43$^{WT/WT}$ (purple) mice. This suggests that transport disruption occurs between 1.5 and 3 months. Presented 1.5 month data are not significantly different from NTg control (not shown).

*$p < 0.05$, **$p < 0.01$, and ***$p < 0.001$, Dunnett's/Dunn's multiple-comparisons test. NS, not significant, unpaired t test/Mann-Whitney U test. n = 5–10. Means ± SEM are plotted for all graphs.

See also Figures S2 and S4.

aged 3–18 months. There were no significant changes in cargo dynamics (Figure S4), suggesting that the mild pausing defect of 18-month-old mutant Fus mice is unlikely to be a direct consequence of aging and that axonal transport of signaling endosomes remains unaltered in wild-type mice up to 18 months.

DISCUSSION

Here, we show that an ALS mouse model of mutant TDP-43 displays a pre-symptomatic, *in vivo* deficit in axonal transport of signaling endosomes in peripheral axons, which may contribute to motor function deficits and impaired neuromuscular integrity. This defect is specific to the M337V mutation, as TDP-43^{WT} protein, which is expressed at a similar, low level as TDP-43^{M337V} relative to endogenous mouse TDP-43 (Gordon et al., 2019), had no effect on retrograde transport rates. Counter to observations of several, but not all (Gordon et al., 2019), reported TDP-43-linked pathologies (Fratta et al., 2018; White et al., 2018), hemizygous and homozygous TDP-43 mutant mice show similar deficiencies in trafficking, suggesting that once a threshold level of mutant TDP-43 is present, no further transport exacerbation occurs. This may be due to the heterozygous mutant TDP-43 transport defect being caused by a loss of signaling or alterations of the axonal proteome that precipitate a maximum physiological reduction in endosome transport speeds, which cannot be further affected by additional pathological protein. Furthermore, once manifested, the transport defect does not appear to get

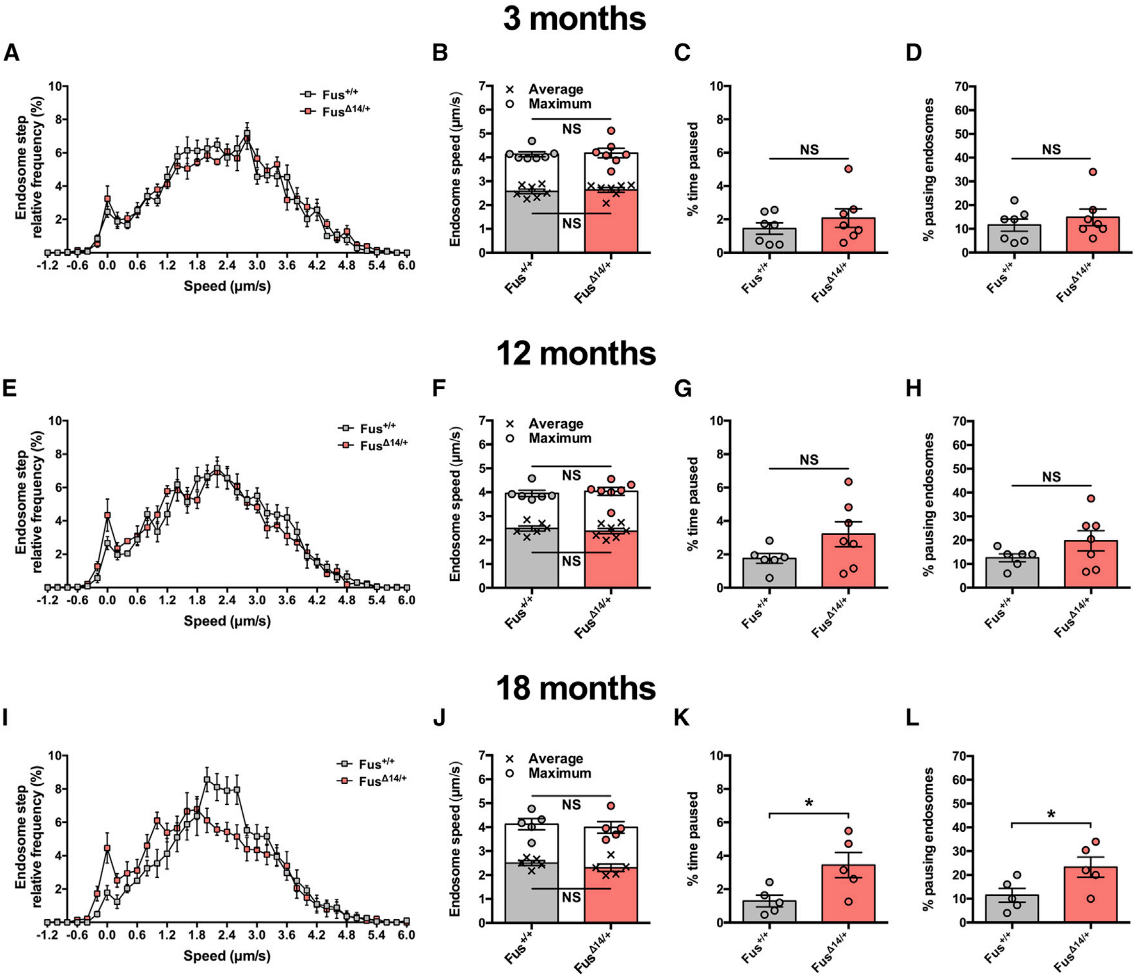

Figure 4. Fus$^{\Delta14/+}$ Mice Display a Minor Impairment in Axonal Transport of Endosomes, but Only at a Late Disease Stage

(A–H) The axonal dynamics of signaling endosomes are similar between Fus$^{+/+}$ (gray) and Fus$^{\Delta14/+}$ (red) mice at 3 months (A–D) and 12 months (E–H) of age.

(I–L) At 18 months, retrograde axonal transport speed of endosomes is unaffected in Fus$^{\Delta14/+}$ mice (I and J); however, mutant Fus mice do show a significant increase in the percentage of time that endosomes pause for (K) and the percentage of endosomes that paused (L).

*$p < 0.05$; NS, not significant, unpaired t test. n = 5–7. Means ± SEM are plotted for all graphs.

See also Figures S3 and S4.

progressively worse between 3 and 9 months, indicating that a disturbance in retrograde transport of signaling endosomes may underlie the subsequent progressive reduction in neuromuscular integrity and motor function, yet is compatible with motor neuron survival at the level of the spinal cord (Gordon et al., 2019).

Alterations in the population of sciatic nerve axons over the imaging period are unlikely to account for the different rates of TDP-43^{M337V} transport, as there is no spinal cord motor neuron loss at the time points assessed (Gordon et al., 2019). Importantly, loss of neuromuscular junction connectivity does not occur until after 3 months, once again indicating that altered sciatic nerve axon profiles do not cause the transport disruption. Furthermore, loss of motor axons does not necessarily result in impaired axonal endosome dynamics, as revealed by our experiments in the Fus$^{\Delta14/+}$ mouse, likely reflecting the robustness of our technique to accurately differentiate between motor and sensory axons. All genotypes assessed showed plentiful uptake and retrograde transport of the fluorescent probe, with no overt changes in endosome number between mutant and wild-type mice, despite endocytosis dysfunction being previously linked to both TDP-43 and FUS toxicity (Liu et al., 2020). Moreover, heterogeneity in axonal endosome-associated Rab proteins, in particular Rab5 and Rab7, is unlikely to cause the observed transport distinctions because Rab7 is the predominant Rab associated with H_CT-positive axonal endosomes (Deinhardt et al., 2006).

Nonetheless, the TDP-43^{M337V} mouse data add to the impaired mitochondrial transport reported in TDP-43^{A315T} mice and defective mitochondria and signaling endosome trafficking in SOD1^{G93A} mice (Table S1). ALS-linked mutations in *SOD1* and *TARDBP* may thus cause early/pre-symptomatic, generalized defects in axonal transport in motor neurons (rather than cargo-specific deficits), leading to dysfunction and degeneration

(Gordon et al., 2019). This may be caused by cargo-independent impairments in the cytoskeleton or motor proteins (e.g., the cytoskeletal regulator HDAC6 is a known target of TDP-43; Fiesel et al., 2010), or by aberrant binding of mutant ALS proteins to motor complexes (Tateno et al., 2009; Zhang et al., 2007); however, this will have to be directly confirmed in the TDP-43^{M337V} model.

Contrastingly, signaling endosome transport in Fus$^{\Delta14/+}$ mice remained largely unaffected even during latter disease stages, despite a 20% loss of spinal cord motor neurons at 18 months (Devoy et al., 2017), confirming that degenerating axons do not always have altered transport kinetics (Malik et al., 2011). This implies that transport disturbances are not necessarily a non-specific by-product of neurodegeneration, at least during earlier disease stages, and thus emphasizes the specificity of transport defects in mutant TDP-43 and SOD1 mice. However, it remains possible that motor neuron loss proceeds very rapidly and targets only specific subpools of motor neurons in Fus$^{\Delta14/+}$ mice, such that any preceding defect in transport was missed (Nijssen et al., 2017). Alternatively, mutant Fus mice may display cargo-specific (e.g., mitochondria, RNA granules) or anterograde transport defects, some of which have been reported in other ALS models (Alami et al., 2014; Baldwin et al., 2016), thus additional cargoes should also be assessed in Fus$^{\Delta14/+}$ mice. Nevertheless, altogether our findings indicate that pre-symptomatic abnormalities in retrograde axonal transport of neurotrophin-containing signaling endosomes may not be common to all ALS-linked genes and that there are inherent distinctions in the pathomechanism of ALS caused by mutations in different RNA processing genes. Although TDP-43 and FUS are both RNA/DNA-binding proteins that process RNA predominantly in the nucleus, they regulate the expression and splicing of largely distinct gene sets (Colombrita et al., 2012; Lagier-Tourenne et al., 2012) and show neuropathological idiosyncrasies when mutated (Bäumer et al., 2010), which could account for these discrepancies in axonal transport deficits. As could the observation that wild-type TDP-43 and SOD1 proteins consistently associate with motor neuron signaling endosomes, whereas FUS does not (Debaisieux et al., 2016).

Disruptions in axonal transport have been linked to the *M337V TARDBP* mutation in a range of *in vitro* and *Drosophila* larval models (Alami et al., 2014; Baldwin et al., 2016; Wang et al., 2013). Although the severe frameshift *FUS* mutation modeled in Fus$^{\Delta14/+}$ mice has not previously been assessed, transport perturbations have been reported in several mutant *FUS* models, including *Drosophila* larvae (Baldwin et al., 2016), isolated squid axoplasm (Sama et al., 2017), and human motor neurons derived from induced pluripotent stem cells (iPSCs) (Guo et al., 2017).

Why then do Fus$^{\Delta14/+}$ mice not show impaired signaling endosome transport, at least until a late disease stage? In addition to the possibilities mentioned above, there are other potential explanations. First, distinctions may arise because of the different FUS mutations being analyzed and their expression in the presence or absence of the wild-type allele. Second, although *Drosophila* is an excellent model that has provided instrumental insights into neurobiology, as well as neurological diseases (Grice et al., 2011; Walters et al., 2019; Yamaguchi and Takashima, 2018), *in vivo* transport analyses are conducted in larvae in which organs have been removed, so there is considerable disruption to the organism, which is being analyzed during development and is thus perhaps not the best model for age-related neurodegeneration. Moreover, the complex, long-range neurotrophin signaling program is not conserved in *Drosophila*, while mutant ALS transgenes are often overexpressed to above physiological levels, which can induce phenotypes even with wild-type FUS transgenes (Baldwin et al., 2016). *In vitro* axonal transport dynamics differ from *in vivo* trafficking (Bilsland et al., 2010; Gibbs et al., 2016), possibly because of cultured neurons lacking the complete series of necessary cellular and chemical interactions (e.g., myelination and target muscle cells in the case of motor neurons) (Sleigh et al., 2017), which is particularly important for ALS as both cell- and non-cell-autonomous pathomechanisms contribute to disease onset and progression (Nijssen et al., 2017). In addition to variability inherent to iPSC differentiation, it remains unknown how closely motor neuron developmental stages in culture correlate with age-related degeneration *in vivo*. By imaging axonal transport of signaling endosomes in intact sciatic nerves of anesthetized mice, we are instead certain of the disease stage and physiological environment of the peripheral axons under investigation.

In summary, we have assessed *in vivo* retrograde axonal transport of signaling endosomes in two mouse models of ALS that express disease-causing mutant proteins at near endogenous levels. Mutant TDP-43, but not mutant Fus, mice displayed a pre-symptomatic deficiency in endosome transport, suggesting that reduced neurotrophin signaling may contribute to mutant TDP-43-mediated neuropathology and that general defects in axonal transport are specific to a subset of ALS-linked genes in an *in vivo* mammalian setting.

STAR★METHODS

Detailed methods are provided in the online version of this paper and include the following:

- KEY RESOURCES TABLE
- LEAD CONTACT AND MATERIALS AVAILABILITY
- EXPERIMENTAL MODEL AND SUBJECT DETAILS
  - Animals
- METHOD DETAILS
  - Axonal transport imaging
  - Axonal transport analysis
  - Axon calibre analysis
- QUANTIFICATION AND STATISTICAL ANALYSIS
- DATA AND CODE AVAILABILITY

SUPPLEMENTAL INFORMATION

Supplemental Information can be found online at https://doi.org/10.1016/j.celrep.2020.02.078.

ACKNOWLEDGMENTS

We would like to thank Robert M. Brownstone (UCL Queen Square Institute of Neurology, University College London) for sharing the ChAT-eGFP mice. This work was supported by the Wellcome Trust Sir Henry Wellcome Postdoctoral Fellowship (103191/Z/13/Z) (J.N.S.), the Medical Research Council Career

Development Award (MR/S006990/1) (J.N.S.), the Motor Neurone Disease Association (D.G., A.D., P.F., E.M.C.F., and K.T.), the Medical Research Council (A.D., P.F., and E.M.C.F.), the American Amyotrophic Lateral Sclerosis Association (A.D. and E.M.C.F.), the Rosetrees Trust (P.F. and E.M.C.F.), the Wellcome Trust Senior Investigator Award (107116/Z/15/Z) (G.S.), the European Union's Horizon 2020 Research and Innovation Programme under grant agreement 739572 (G.S.), and a UK Dementia Research Institute Foundation award (G.S.).

AUTHOR CONTRIBUTIONS

J.N.S. and A.P.T. performed the research and analyzed the data. D.G., A.D., P.F., E.M.C.F., and K.T. provided mice and expertise. J.N.S., A.P.T., and G.S. wrote the paper with input from all authors. All authors approved submission of this manuscript.

DECLARATION OF INTERESTS

The authors declare no competing interests.

Received: April 5, 2019
Revised: October 11, 2019
Accepted: February 19, 2020
Published: March 17, 2020

SUPPORTING CITATIONS

The following reference appears in the Supplemental Information: Gurney et al., 1994; Wegorzewska et al., 2009.

REFERENCES

Al-Chalabi, A., and Hardiman, O. (2013). The epidemiology of ALS: a conspiracy of genes, environment and time. Nat. Rev. Neurol. *9*, 617–628.

Alami, N.H., Smith, R.B., Carrasco, M.A., Williams, L.A., Winborn, C.S., Han, S.S.W., Kiskinis, E., Winborn, B., Freibaum, B.D., Kanagaraj, A., et al. (2014). Axonal transport of TDP-43 mRNA granules is impaired by ALS-causing mutations. Neuron *81*, 536–543.

Baldwin, K.R., Godena, V.K., Hewitt, V.L., and Whitworth, A.J. (2016). Axonal transport defects are a common phenotype in *Drosophila* models of ALS. Hum. Mol. Genet. *25*, 2378–2392.

Bäumer, D., Hilton, D., Paine, S.M.L., Turner, M.R., Lowe, J., Talbot, K., and Ansorge, O. (2010). Juvenile ALS with basophilic inclusions is a FUS proteinopathy with FUS mutations. Neurology *75*, 611–618.

Bilsland, L.G., Sahai, E., Kelly, G., Golding, M., Greensmith, L., and Schiavo, G. (2010). Deficits in axonal transport precede ALS symptoms *in vivo*. Proc. Natl. Acad. Sci. USA *107*, 20523–20528.

Bronfman, F.C., Escudero, C.A., Weis, J., and Kruttgen, A. (2007). Endosomal transport of neurotrophins: roles in signaling and neurodegenerative diseases. Dev. Neurobiol. *67*, 1183–1203.

Brown, R.H., and Al-Chalabi, A. (2017). Amyotrophic lateral sclerosis. N. Engl. J. Med. *377*, 162–172.

Clark, J.A., Yeaman, E.J., Blizzard, C.A., Chuckowree, J.A., and Dickson, T.C. (2016). A case for microtubule vulnerability in amyotrophic lateral sclerosis: altered dynamics during disease. Front. Cell. Neurosci. *10*, 204.

Colombrita, C., Onesto, E., Megiorni, F., Pizzuti, A., Baralle, F.E., Buratti, E., Silani, V., and Ratti, A. (2012). TDP-43 and FUS RNA-binding proteins bind distinct sets of cytoplasmic messenger RNAs and differently regulate their post-transcriptional fate in motoneuron-like cells. J. Biol. Chem. *287*, 15635–15647.

De Vos, K.J., and Hafezparast, M. (2017). Neurobiology of axonal transport defects in motor neuron diseases: Opportunities for translational research? Neurobiol. Dis. *105*, 283–299.

Debaisieux, S., Encheva, V., Chakravarty, P., Snijders, A.P., and Schiavo, G. (2016). Analysis of signaling endosome composition and dynamics using SILAC in embryonic stem cell-derived neurons. Mol. Cell. Proteomics *15*, 542–557.

Deinhardt, K., Salinas, S., Verastegui, C., Watson, R., Worth, D., Hanrahan, S., Bucci, C., and Schiavo, G. (2006). Rab5 and Rab7 control endocytic sorting along the axonal retrograde transport pathway. Neuron *52*, 293–305.

Devoy, A., Kalmar, B., Stewart, M., Park, H., Burke, B., Noy, S.J., Redhead, Y., Humphrey, J., Lo, K., Jaeger, J., et al. (2017). Humanized mutant FUS drives progressive motor neuron degeneration without aggregation in 'FUSDelta14' knockin mice. Brain *140*, 2797–2805.

Fellows, A.D., Rhymes, E.R., Gibbs, K.L., Greensmith, L., and Schiavo, G. (2020). IGF1R regulates retrograde axonal transport of signalling endosomes in motor neurons. EMBO Rep. Published online February 7, 2020. https://doi.org/10.15252/embr.201949129.

Fiesel, F.C., Voigt, A., Weber, S.S., Van den Haute, C., Waldenmaier, A., Görner, K., Walter, M., Anderson, M.L., Kern, J.V., Rasse, T.M., et al. (2010). Knockdown of transactive response DNA-binding protein (TDP-43) downregulates histone deacetylase 6. EMBO J. *29*, 209–221.

Fratta, P., Sivakumar, P., Humphrey, J., Lo, K., Ricketts, T., Oliveira, H., Brito-Armas, J.M., Kalmar, B., Ule, A., Yu, Y., et al. (2018). Mice with endogenous TDP-43 mutations exhibit gain of splicing function and characteristics of amyotrophic lateral sclerosis. EMBO J. *37*, e98684.

Gibbs, K.L., Kalmar, B., Sleigh, J.N., Greensmith, L., and Schiavo, G. (2016). *In vivo* imaging of axonal transport in murine motor and sensory neurons. J. Neurosci. Methods *257*, 26–33.

Gibbs, K.L., Kalmar, B., Rhymes, E.R., Fellows, A.D., Ahmed, M., Whiting, P., Davies, C.H., Greensmith, L., and Schiavo, G. (2018). Inhibiting p38 MAPK alpha rescues axonal retrograde transport defects in a mouse model of ALS. Cell Death Dis. *9*, 596.

Gordon, D., Dafinca, R., Scaber, J., Alegre-Abarrategui, J., Farrimond, L., Scott, C., Biggs, D., Kent, L., Oliver, P.L., Davies, B., et al. (2019). Single-copy expression of an amyotrophic lateral sclerosis-linked TDP-43 mutation (M337V) in BAC transgenic mice leads to altered stress granule dynamics and progressive motor dysfunction. Neurobiol. Dis. *121*, 148–162.

Grice, S.J., Sleigh, J.N., Liu, J.L., and Sattelle, D.B. (2011). Invertebrate models of spinal muscular atrophy: insights into mechanisms and potential therapeutics. BioEssays *33*, 956–965.

Guo, W., Naujock, M., Fumagalli, L., Vandoorne, T., Baatsen, P., Boon, R., Ordovás, L., Patel, A., Welters, M., Vanwelden, T., et al. (2017). HDAC6 inhibition reverses axonal transport defects in motor neurons derived from FUS-ALS patients. Nat. Commun. *8*, 861.

Gurney, M.E., Pu, H., Chiu, A.Y., Dal Canto, M.C., Polchow, C.Y., Alexander, D.D., Caliendo, J., Hentati, A., Kwon, Y.W., Deng, H.X., et al. (1994). Motor neuron degeneration in mice that express a human Cu,Zn superoxide dismutase mutation. Science *264*, 1772–1775.

Lagier-Tourenne, C., Polymenidou, M., Hutt, K.R., Vu, A.Q., Baughn, M., Huelga, S.C., Clutario, K.M., Ling, S.-C., Liang, T.Y., Mazur, C., et al. (2012). Divergent roles of ALS-linked proteins FUS/TLS and TDP-43 intersect in processing long pre-mRNAs. Nat. Neurosci. *15*, 1488–1497.

Liu, G., Byrd, A., Warner, A.N., Pei, F., Basha, E., Buchanan, A., and Buchan, J.R. (2020). Cdc48/VCP and endocytosis regulate TDP-43 and FUS toxicity and turnover. Mol. Cell. Biol. *40*, e00256-19.

Maday, S., Twelvetrees, A.E., Moughamian, A.J., and Holzbaur, E.L.F. (2014). Axonal transport: cargo-specific mechanisms of motility and regulation. Neuron *84*, 292–309.

Magrané, J., Cortez, C., Gan, W.-B., and Manfredi, G. (2014). Abnormal mitochondrial transport and morphology are common pathological denominators in SOD1 and TDP43 ALS mouse models. Hum. Mol. Genet. *23*, 1413–1424.

Malik, B., Nirmalananthan, N., Bilsland, L.G., La Spada, A.R., Hanna, M.G., Schiavo, G., Gallo, J.-M., and Greensmith, L. (2011). Absence of disturbed axonal transport in spinal and bulbar muscular atrophy. Hum. Mol. Genet. *20*, 1776–1786.

Mohan, R., Tosolini, A.P., and Morris, R. (2014). Targeting the motor end plates in the mouse hindlimb gives access to a greater number of spinal cord motor

neurons: an approach to maximize retrograde transport. Neuroscience *274*, 318–330.

Nijssen, J., Comley, L.H., and Hedlund, E. (2017). Motor neuron vulnerability and resistance in amyotrophic lateral sclerosis. Acta Neuropathol. *133*, 863–885.

Renton, A.E., Chiò, A., and Traynor, B.J. (2014). State of play in amyotrophic lateral sclerosis genetics. Nat. Neurosci. *17*, 17–23.

Restani, L., Giribaldi, F., Manich, M., Bercsenyi, K., Menendez, G., Rossetto, O., Caleo, M., and Schiavo, G. (2012). Botulinum neurotoxins A and E undergo retrograde axonal transport in primary motor neurons. PLoS Pathog. *8*, e1003087.

Sama, R.R.K., Fallini, C., Gatto, R., McKeon, J.E., Song, Y., Rotunno, M.S., Penaranda, S., Abdurakhmanov, I., Landers, J.E., Morfini, G., et al. (2017). ALS-linked FUS exerts a gain of toxic function involving aberrant p38 MAPK activation. Sci. Rep. *7*, 115.

Sleigh, J., and Schiavo, G. (2016). Older but not slower: aging does not alter axonal transport dynamics of signalling endosomes in vivo. Matters. https://sciencematters.io/articles/201605000018.

Sleigh, J.N., Vagnoni, A., Twelvetrees, A.E., and Schiavo, G. (2017). Methodological advances in imaging intravital axonal transport. F1000Res. *6*, 200.

Sleigh, J.N., Rossor, A.M., Fellows, A.D., Tosolini, A.P., and Schiavo, G. (2019). Axonal transport and neurological disease. Nat. Rev. Neurol. *15*, 691–703.

Surana, S., Tosolini, A.P., Meyer, I.F.G., Fellows, A.D., Novoselov, S.S., and Schiavo, G. (2018). The travel diaries of tetanus and botulinum neurotoxins. Toxicon *147*, 58–67.

Tateno, M., Kato, S., Sakurai, T., Nukina, N., Takahashi, R., and Araki, T. (2009). Mutant SOD1 impairs axonal transport of choline acetyltransferase and acetylcholine release by sequestering KAP3. Hum. Mol. Genet. *18*, 942–955.

Tosolini, A.P., and Sleigh, J.N. (2017). Motor neuron gene therapy: lessons from spinal muscular atrophy for amyotrophic lateral sclerosis. Front. Mol. Neurosci. *10*, 405.

Villarroel-Campos, D., Schiavo, G., and Lazo, O.M. (2018). The many disguises of the signalling endosome. FEBS Lett. *592*, 3615–3632.

Walters, R., Manion, J., and Neely, G.G. (2019). Dissecting motor neuron disease with *Drosophila melanogaster*. Front. Neurosci. *13*, 331.

Wang, W., Li, L., Lin, W.-L., Dickson, D.W., Petrucelli, L., Zhang, T., and Wang, X. (2013). The ALS disease-associated mutant TDP-43 impairs mitochondrial dynamics and function in motor neurons. Hum. Mol. Genet. *22*, 4706–4719.

Wegorzewska, I., Bell, S., Cairns, N.J., Miller, T.M., and Baloh, R.H. (2009). TDP-43 mutant transgenic mice develop features of ALS and frontotemporal lobar degeneration. Proc. Natl. Acad. Sci. U S A *106*, 18809–18814.

White, M.A., Kim, E., Duffy, A., Adalbert, R., Phillips, B.U., Peters, O.M., Stephenson, J., Yang, S., Massenzio, F., Lin, Z., et al. (2018). TDP-43 gains function due to perturbed autoregulation in a *Tardbp* knock-in mouse model of ALS-FTD. Nat. Neurosci. *21*, 552–563.

Williamson, M.G., Finelli, M.J., Sleigh, J.N., Reddington, A., Gordon, D., Talbot, K., Davies, K.E., and Oliver, P.L. (2019). Neuronal over-expression of Oxr1 is protective against ALS-associated mutant TDP-43 mislocalisation in motor neurons and neuromuscular defects *in vivo*. Hum. Mol. Genet. *28*, 3584–3599.

Yamaguchi, M., and Takashima, H. (2018). *Drosophila* Charcot-Marie-Tooth disease models. Adv. Exp. Med. Biol. *1076*, 97–117.

Zhang, F., Ström, A.-L., Fukada, K., Lee, S., Hayward, L.J., and Zhu, H. (2007). Interaction between familial amyotrophic lateral sclerosis (ALS)-linked SOD1 mutants and the dynein complex. J. Biol. Chem. *282*, 16691–16699.

STAR★METHODS

KEY RESOURCES TABLE

REAGENT or RESOURCE	SOURCE	IDENTIFIER
Chemicals, Peptides, and Recombinant Proteins		
H_CT^{441}	Restani et al., 2012	N/A
AlexaFluor555 C_2 maleimide	Life Technologies	A-20346
recombinant human brain-derived neurotrophic factor	Peprotech	450-02
Experimental Models: Organisms/Strains		
Mouse: Tg(Chat-EGFP)GH293Gsat/Mmucd (ChAT-eGFP)	Mutant Mouse Resource and Research Center	MMRRC: 000296-UCD
Mouse: B6;129S6-Gt(ROSA)26Sor$^{m1(TARDBP*M337V/Ypet)Tlbt}$/J (WT and M337V TDP43)	The Jackson Laboratory	JAX: 029266
Mouse: B6N;B6J-Fus$^{tm1Emcf./H}$ (Fus$^{+/+}$ and Fus$^{\Delta14/+}$)	Devoy et al., 2017	N/A
Software and Algorithms		
Tracker (Version 2.0.0.26)	Kinetic Imaging Ltd.	N/A

LEAD CONTACT AND MATERIALS AVAILABILITY

Further information and requests for resources and reagents should be directed to and will be fulfilled by the Lead Contact, Giampietro Schiavo (giampietro.schiavo@ucl.ac.uk). This study did not generate new unique reagents.

EXPERIMENTAL MODEL AND SUBJECT DETAILS

Animals

All mouse handling and experiments were performed under license from the United Kingdom Home Office in accordance with the Animals (Scientific Procedures) Act (1986) and approved by the University College London – Queen Square Institute of Neurology Ethics Committee. All mice were maintained in individually ventilated cages and on a standard diet. Tg(Chat-EGFP) GH293Gsat/Mmucd mice (MMRRC Stock Number 000296-UCD), referred to as ChAT-eGFP mice, were maintained and imaged as heterozygotes on a CD-1 background. The male to female ratio used for these experiments was 2:3. B6;129S6-Gt(ROSA)26Sor$^{m1(TARDBP*M337V/Ypet)Tlbt}$/J (WT and M337V TDP43, Jackson Laboratory strain #029266, https://www.jax.org/strain/029266) and B6N;B6J-Fus$^{tm1Emcf./H}$ (Fus$^{+/+}$ and Fus$^{\Delta14/+}$) mice were maintained on a C57BL/6 background and genotyped as detailed (Devoy et al., 2017; Gordon et al., 2019). ChAT-eGFP mice used for motor versus sensory analyses were 79-134 days old. Non-transgenic (NTg) control and TDP43 mice sacrificed for 1.5, 3, and 9 month time points were 55-57, 102-125, and 249-71 days old, respectively. The male to female ratio used for these time points were 1:1, 2:15 and 35:6, respectively. Fus mice sacrificed for 3, 12, and 18 month time points were 104-115, 365-368, and 568-588 days old, respectively. The male to female ratio used for these time points were 4:3, 6:7 and 3:7, respectively. Littermates of either sex were pooled in all analyses because no significant differences in transport were observed between males and females independent of the genotype (Figures S4E–S4H, and data not shown). Sample sizes of 5 or above were chosen based on two-sample, two-sided power calculations, with standard power of 0.8 ($1-\beta$) and type I error rate of 5% (α). Estimated mean and standard deviation of average signaling endosome transport speeds per animal were calculated from previous data generated from mice modeling neuromuscular diseases.

METHOD DETAILS

Axonal transport imaging

In vivo kinetics of signaling endosomes labeled with atoxic binding fragment of tetanus neurotoxin (H_CT) were assessed as previously described (Gibbs et al., 2016; J.N.S., A.P.T., and G.S., unpublished data). Briefly, H_CT (H_CT^{441}, residues 875-1315) fused to an improved cysteine-rich tag and a human influenza haemagglutinin epitope was bacterially expressed as a glutathione-*S*-transferase fusion protein (Restani et al., 2012), and labeled with AlexaFluor555 C_2 maleimide (Life Technologies, A-20346). On the morning of analysis, H_CT was pre-mixed with recombinant human brain-derived neurotrophic factor (BDNF, Peprotech, 450-02) in phosphate buffered saline. Under isofluorane-induced anesthesia and on a heat-pad to maintain body temperature, two 1-2 mm long incisions were made in the skin above the gastrocnemius and tibialis anterior muscles of the right leg. 3.5-5 μg of H_CT with 25 ng BDNF was

then injected in a volume of ≈1.5 μL per muscle into the motor end plate region as per Mohan et al. (2014). The incisions were closed by suturing and the animal allowed to fully recover. 4-8 h post-injection, animals were terminally anaesthetised using isofluorane and 1–1.5 cm of the right sciatic nerve was exposed by removal of the overlying skin and musculature. Curved forceps were then used to cautiously separate the sciatic nerve from the underlying connective tissue, such that a small piece of magic tape could be placed between the two to aid imaging. Still under anesthesia, the mouse was transferred to an inverted LSM780 laser scanning microscope (Zeiss) within an environmental chamber pre-warmed and set throughout the experiment to 37°C. The body weight of the animal and careful positioning ensures that the sciatic nerve remains stationary on the coverslip overlying the 63x Plan-Apochromat oil immersion objective lens (Zeiss). An area containing several axons retrogradely transporting the fluorescent H_CT probe was selected and imaged every 2.4-3.2 s at 100x digital zoom (1024x1024, 1% laser power). All imaging was completed within 1 h of initiating terminal anesthesia.

Axonal transport analysis

Confocal image series were converted into .avi files and individual endosome dynamics manually tracked using Tracker (Kinetic Imaging Ltd.) (Figure S1A). Endosomes were included in the analysis if they could be observed for $\geq$ 5 consecutive frames and did not pause for > 10 consecutive images. Endosomes that were tracked and then paused for long periods were not included for fear of issues associated with phototoxicity. Nevertheless, on average fewer than one endosome per animal did this and the phenotype was not linked to a particular genotype. Moreover, endosomes moving solely in the anterograde direction were also not included as they were similarly infrequent. All individual frame-to-frame step speeds are included in the presented speed frequency histograms (459.6 ± 11.4 frame-to-frame speeds per animal were calculated), meaning that an endosome tracked across 11 consecutive frames will generate 10 frame-to-frame speeds to be included in the frequency histogram. To determine the mean endosome speed per animal, the speeds of individual endosomes were calculated and then an average of these speeds determined (50.3 ± 0.8 endosomes per animal were tracked). The fastest endosome speed per animal is reported as the 'maximum speed'. All speed analyses include frames and time during which endosomes may have been paused, i.e., we report the speed across the entire tracked run length and not the speed solely when motile. An endosome was considered to have paused if it remained in the same position for two consecutive images. The '% time paused' is a calculation of the length of time all tracked endosomes remained stationary, while the '% pausing endosomes' details the proportion of endosomes that displayed at least one pause while being tracked. At least six endosomes from at least two individual, thick axons were assessed per animal.

Axon calibre analysis

Axon calibres were determined from images taken for endosome transport analyses by measuring the distance between the upper and lower margins of transported fluorescent signaling endosomes orthogonally from the direction of transport. A minimum of ten measurements were made along the length of the axon to calculate average widths per axon, and three different axons per animal were used to calculate a per animal mean width.

QUANTIFICATION AND STATISTICAL ANALYSIS

Data were assumed to be normally distributed unless evidence to the contrary could be provided by the D'Agostino and Pearson omnibus normality test. Normally distributed data were statistically analyzed using a t test or one-way analysis of variance (ANOVA) with Dunnett's multiple comparisons test, and non-normally distributed data with a Mann-Whitney *U* test or Kruskal-Wallis test with Dunn's multiple comparisons test. Paired t tests were used to compare transport kinetics in $ChAT^+$ versus $ChAT^-$ axons as data were generated from the same animals. Endosomes were tracked from videos in which the genotype of the animal was blinded. All tests were two-tailed and an α-level of $p < 0.05$ was used to determine significance. GraphPad Prism 6 software was used for all statistical analyses and figure production. All figure legends contain details of statistical tests and sample sizes (*i.e*. number of animals) used, with dispersion and precision measures.

DATA AND CODE AVAILABILITY

This study did not generate/analyze datasets/code.

Cell Reports, Volume *30*

Supplemental Information

Mice Carrying ALS Mutant TDP-43,

but Not Mutant FUS, Display *In Vivo* Defects

in Axonal Transport of Signaling Endosomes

James N. Sleigh, Andrew P. Tosolini, David Gordon, Anny Devoy, Pietro Fratta, Elizabeth M.C. Fisher, Kevin Talbot, and Giampietro Schiavo

Supplemental Figures

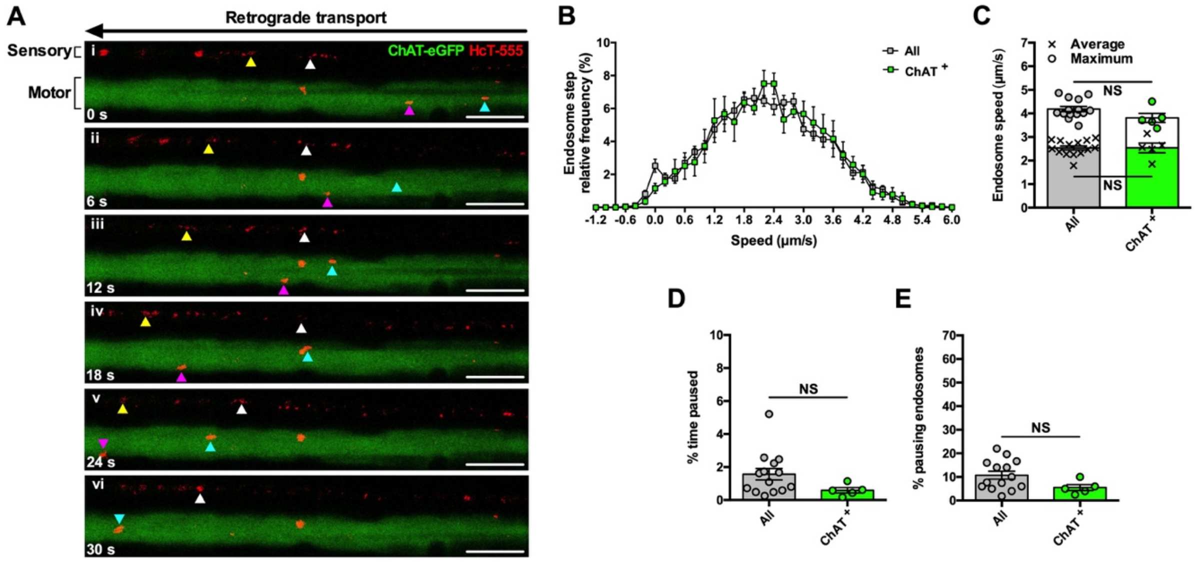

Figure S1. Signalling endosome dynamics are similar between ChAT$^+$ axons and the neurons assessed in non-fluorescent animals. Related to Figure 1. (**A**) Individual signalling endosomes loaded with fluorescently labelled HcT (HcT-555, red) can be imaged in live, intact sciatic nerve axons using time-lapse confocal microscopy and their dynamics subsequently assessed. This series of images (i-vi) depicts retrograde (right to left) axonal transport of distinct endosomes identified by coloured arrowheads. These images were taken from a ChAT-eGFP mouse, which allows the differentiation between motor (ChAT-eGFP$^+$, green) and sensory (ChAT-eGFP$^-$) axons. Note how much thinner the sensory axons are than the motor axons, causing endosome transport in sensory nerves to occur in a narrow, straight line. Scale bars = 10 μm. (**B**) The speed distribution curve of signalling endosome frame-to-frame movements in ChAT$^+$ motor neurons (green) is similar to that generated from all axons of 3 month old wild-type, non-fluorescent mice (All, grey). This suggests that motor neurons are predominantly assessed throughout this study. (**C-E**) There is no difference between ChAT$^+$ motor neurons and axons from 3 month old, non-fluorescent wild-type mice in mean endosome speeds (crosses, C), maximum endosome speeds (circles, C), percentage time paused (D), or the percentage of endosomes that paused (E). The ChAT$^+$ data presented here are the same as that in **Figure 1**. NS, not significant, unpaired *t*-test. *n* = 5-14. Means ± SEM are plotted for all graphs.

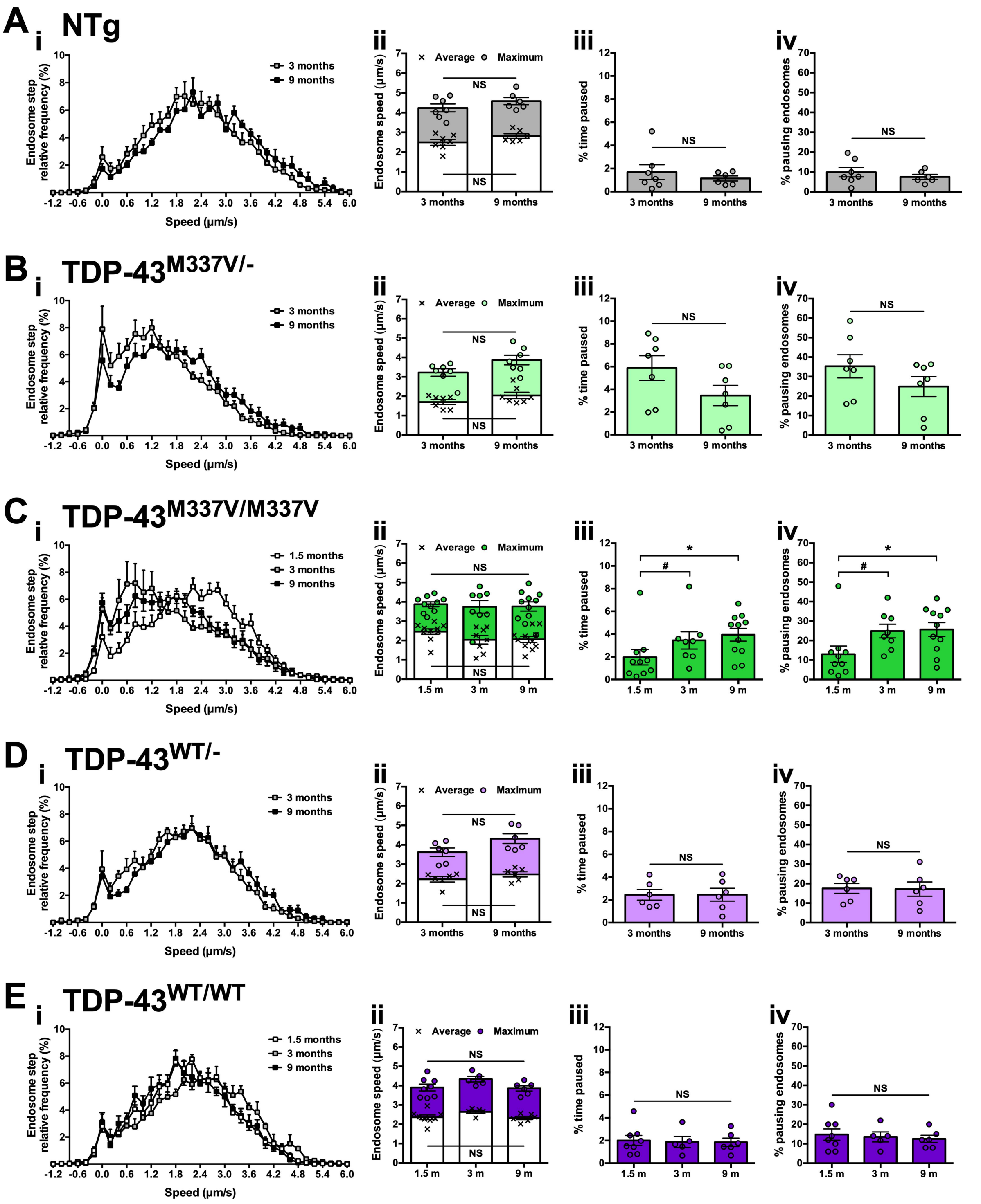

Figure S2. The TDP-43^{M337V} transport defect presents between 1.5 and 3 months and persists thereafter. Related to Figures 2 and 3. (**A-E**) Axonal signalling endosome kinetics do not change significantly from 3 to 9 months in NTg (A), TDP-43$^{M337V/-}$ (B), and TDP-43$^{WT/-}$ (D) mice or from 1.5 to 9 months in TDP-43$^{WT/WT}$ animals (E); however, signalling endosome transport becomes impaired in TDP-43$^{M337V/M337V}$ mice between 1.5 and 3 months (C, ii $P = 0.143$; iii $P = 0.0273$; iv $P = 0.0209$, Kruskal-Wallis test). The data presented in this figure are also found in **Figures 2** and **3**. $^{\#}$ $P < 0.05$; Mann-Whitney *U* test; * $P < 0.05$; Dunn's multiple comparisons test. NS, not significant, unpaired *t*-test/Mann Whitney *U* test/one-way ANOVA. n = 5-11. Means ± SEM are plotted for all graphs.

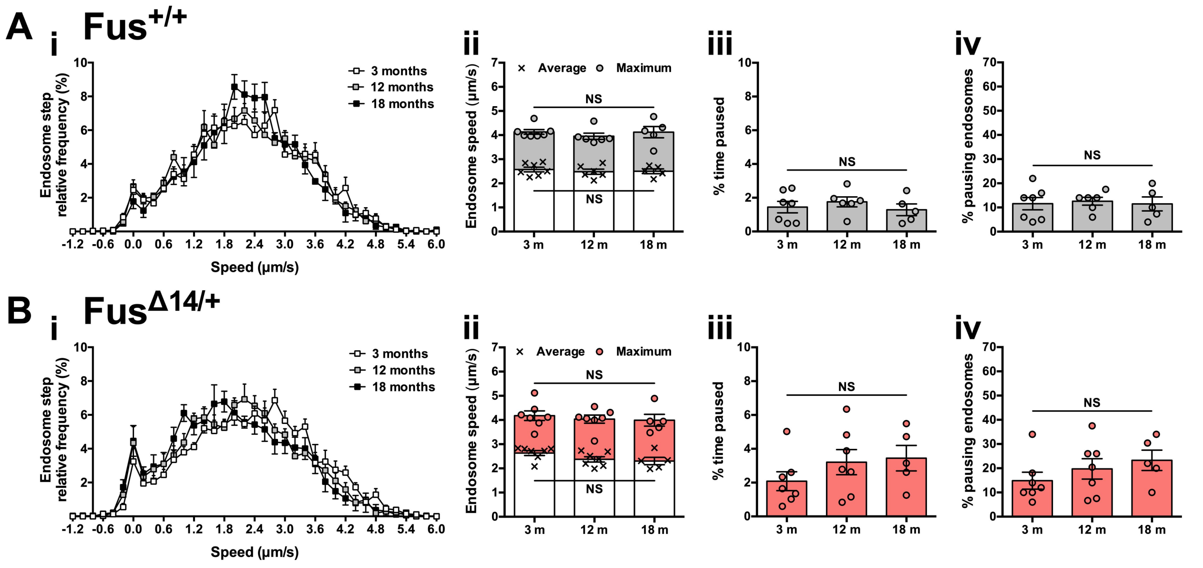

Figure S3. Fus$^{\Delta14/+}$ axonal transport remains largely unchanged between 3 and 18 months. Related to Figure 4. (**A** and **B**) Endosome axonal dynamics do not significantly change from 3 to 18 months in Fus$^{+/+}$ (A) and Fus$^{\Delta14/+}$ (B) mice. However, there is a minor, progressive decline in endosomal dynamics in mutant Fus mice. The data presented in this figure are also found in **Figure 4**. NS, not significant, one-way ANOVA. n = 5-7. Means ± SEM are plotted for all graphs.

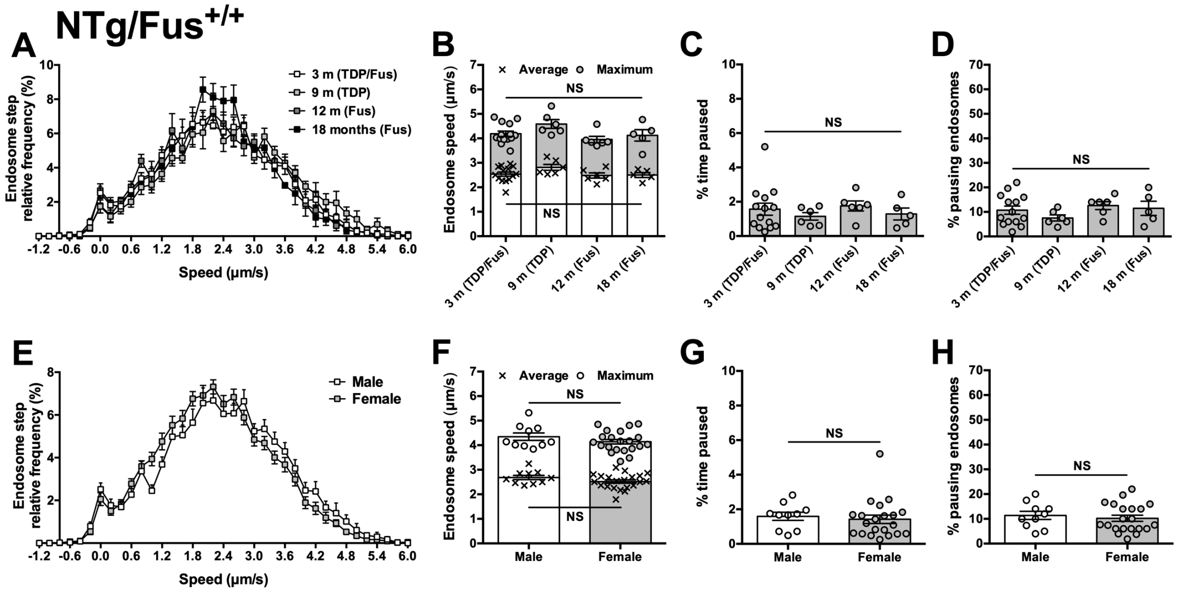

Figure S4. Axonal endosome kinetics do not change from 3 to 18 months or differ between sexes in wild-type mice. Related to Figures 2-4 (**A-D**) There is no difference in signalling endosome dynamics between wild-type mice aged 3, 9, 12, and 18 months, indicating that aging of mice to 18 months does not affect axonal transport of signalling endosomes. Data generated from 3 month old wild-type mice, used as controls for TDP-43^{M337V} and Fus$^{\Delta 14/+}$, were combined as they showed no significant difference (not shown). (**E-H**) There is no difference in axonal kinetics of signalling endosomes between male and female mice. Since there was no difference between timepoints (A-D), combined data from all ages are presented. The data presented in this figure are also found in **Figures 2-4**. NS, not significant, one-way ANOVA. *n* = 5-14 (A-D) and 10-21 (E-H). Means ± SEM are plotted for all graphs.

Supplemental Tables

ALS mouse model	*In vivo* axonal transport	Lumbar motor neuron loss
SOD1^{G93A} [(1)]	Motor neuron-specific, pre-symptomatic (P36 onwards, and worst at P73) reduction in retrograde transport speed of signalling endosomes in sciatic nerve axons (only females tested) [(2-4)]<br><br>Pre-symptomatic (P36) increase in anterograde and retrograde mitochondrial pausing resulting in reduced speeds in sciatic nerve axons (only females tested) [(2)]<br><br>Reduced retrograde mitochondrial movement in sciatic nerve axons at P45, but not P15, progressively worsening and also affecting anterograde transport by P90 (males and females) [(5)]	0% at P36;<br>23% at P73;<br>39% at P94;<br>56% at P113 [(2)]
Prp-TDP-43^{A315T} [(6)]	Reduced anterograde and retrograde mitochondrial movement in sciatic nerve axons at P90, but not P45 (males and females) [(5)]	20% at end stage [(6)]
TDP-43^{M337V} [(7)]	Pre-symptomatic (3 and 9 months, but not 1.5 months) reduction in retrograde transport speed of signalling endosomes in sciatic nerve axons, at least in part, through increased pausing (males and females) [(8)]	0% at 12 months [(7)]
Fus$^{\Delta14/+}$ [(9)]	Retrograde transport of signalling endosomes in sciatic nerve axons unaffected at 3 and 12 months, with only a minor increase in pausing at 18 months, which does not affect transport speed (males and females) [(8)]	0% at 3 months;<br>14% at 12 months;<br>20% at 18 months [(9)]

Table S1. *In vivo* axonal transport phenotypes and motor neuron loss in mouse models of ALS. Related to Figures 2-4. *In vivo* axonal transport defects, as assessed by individual cargo tracking, have now been reported in a number of mouse models of ALS. For comparison purposes, this table provides details on the axonal trafficking disruption and lumbar spinal cord motor neuron loss. *N.B.*, *in vivo* studies in which transport has been assessed *en masse* are not included. [(1)] Gurney et al., 1994; [(2)] Bilsland et al., 2010; [(3)] Gibbs et al., 2018; [(4)] Fellows et al., 2020; [(5)] Magrané et al., 2014; [(6)] Wegorzewska et al., 2009; [(7)] Gordon et al., 2019; [(8)] this study; [(9)] Devoy et al., 2017.
